# DO IT Trial: vitamin D Outcomes and Interventions in Toddlers **–** a TARGet Kids! randomized controlled trial

**DOI:** 10.1186/1471-2431-14-37

**Published:** 2014-02-08

**Authors:** Jonathon L Maguire, Catherine S Birken, Mark B Loeb, Muhammad Mamdani, Kevin Thorpe, Jeffrey S Hoch, Tony Mazzulli, Cornelia M Borkhoff, Colin Macarthur, Patricia C Parkin

**Affiliations:** 1 The Applied Health Research Centre of the Li Ka Shing Knowledge Institute of St. Michael’s Hospital, University of Toronto, Toronto, Ontario, Canada; 2 Department of Pediatrics, St. Michael’s Hospital, 30 Bond Street, 15-014 Cardinal Carter, Toronto, Ontario, M5B 1 W8, Canada; 3 Pediatric Outcomes Research Team (PORT), Division of Pediatric Medicine, Department of Pediatrics, The Hospital for Sick Children, Toronto, Ontario, Canada; 4 Department of Pediatrics, Faculty of Medicine, University of Toronto, Toronto, Ontario, Canada; 5 Leslie Dan Faculty of Pharmacy, University of Toronto, Toronto, Ontario, Canada; 6 Dalla Lana School of Public Health, University of Toronto, Toronto, Ontario, Canada; 7 Institute for Health Policy, Management and Evaluation, University of Toronto, Toronto, Ontario, Canada; 8 Department of Pathology and Molecular Medicine and Clinical Epidemiology and Biostatistics, McMaster University, Hamilton, Ontario, Canada; 9 Pharmacoeconomics Research Unit, Cancer Care Ontario, Toronto, Ontario, Canada; 10 Centre for Excellence in Economic Analysis Research, Li Ka Shing Knowledge Institute of St. Michael’s Hospital, University of Toronto, Toronto, Ontario, Canada; 11 Department of Laboratory Medicine and Pathobiology, Faculty of Medicine, University of Toronto, Toronto, Ontario, Canada; 12 Department of Microbiology, Mount Sinai Hospital and University Health Network, Toronto, Ontario, Canada

**Keywords:** Vitamin D deficiency, Vitamin D supplementation, Infant, Toddler

## Abstract

**Background:**

Vitamin D levels are alarmingly low (<75 nmol/L) in 65-70% of North American children older than 1 year. An increased risk of viral upper respiratory tract infections (URTI), asthma-related hospitalizations and use of anti-inflammatory medication have all been linked with low vitamin D. No study has determined whether wintertime vitamin D supplementation can reduce the risk of URTI and asthma exacerbations, two of the most common and costly illnesses of early childhood. The objectives of this study are: 1) to compare the effect of ‘high dose’ (2000 IU/day) vs. ‘standard dose’ (400 IU/day) vitamin D supplementation in achieving reductions in laboratory confirmed URTI and asthma exacerbations during the winter in preschool-aged Canadian children; and 2) to assess the effect of ‘high dose’ vitamin D supplementation on vitamin D serum levels and specific viruses that cause URTI.

**Methods/Design:**

This study is a pragmatic randomized controlled trial. Over 4 successive winters we will recruit 750 healthy children 1–5 years of age. Participating physicians are part of a primary healthcare research network called *TARGet Kids!*. Children will be randomized to the ‘standard dose’ or ‘high dose’ oral supplemental vitamin D for a minimum of 4 months (200 children per group). Parents will obtain a nasal swab from their child with each URTI, report the number of asthma exacerbations and complete symptom checklists. Unscheduled physician visits for URTIs and asthma exacerbations will be recorded. By May, a blood sample will be drawn to determine vitamin D serum levels. The primary analysis will be a comparison of URTI rate between study groups using a Poisson regression model. Secondary analyses will compare vitamin D serum levels, asthma exacerbations and the frequency of specific viral agents between groups.

**Discussion:**

Identifying whether vitamin D supplementation of preschoolers can reduce wintertime viral URTIs and asthma exacerbations and what dose is optimal may reduce population wide morbidity and associated health care and societal costs. This information will assist in determining practice and health policy recommendations related to vitamin D supplementation in healthy Canadian preschoolers.

## Background

Evidence from observational studies suggests that low vitamin D levels may be implicated in two of the most common health issues during early childhood: viral upper respiratory tract infections (URTI) and asthma [1]. These two conditions place an enormous burden on Canada’s health care system and economy. Viral URTI and asthma exacerbations combined make up over 30% of all emergency department visits for children in Canada [2].

Data from our group [3] and others have repeatedly demonstrated that most urban preschoolers living in North America have vitamin D serum levels significantly lower than values recommended by both the American Academy of Pediatrics (AAP) and the Canadian Paediatric Society (CPS) [4,5]. However, these guidelines are not based on child health outcome data but on expert opinion and extrapolation from adult outcomes. There are no Canadian recommendations for vitamin D supplementation of children older than 1 year. Furthermore, it is not known whether vitamin D supplementation leads to measurable improvement in child health outcomes or what dose (or what vitamin D serum level) is needed to maximize health outcomes in preschoolers.

Because of Canada’s northern latitude, understanding vitamin D deficiency and associated health problems is of critical importance to Canadian children and their parents. Our goal is to close the key knowledge gaps in the potential health consequences associated with low vitamin D levels in young children [4-8]. We are currently conducting a randomized controlled trial to test the effect of high dose orally supplemented vitamin D (2000 IU/day) versus standard dose vitamin D supplementation (400 IU/day) during the wintertime on these common childhood health outcomes in Canadian preschoolers. If vitamin D supplementation of preschoolers makes even a small contribution to improving these problems, measures to increase vitamin D levels may significantly reduce population wide morbidity and associated health service costs.

We have developed a primary-care practice based research network called *TARGet Kids!* to conduct observational and interventional studies in preschoolers to improve child health outcomes through primary prevention. *TARGet Kids!* is first primary care child health research network in Canada dedicated to improving the health of young children. *TARGet Kids!* represents an innovative collaboration between child health researchers in the Faculty of Medicine at the University of Toronto and children’s primary care physicians (pediatricians and family physicians) from the Department of Pediatrics and the Department of Family and Community Medicine at the University of Toronto to advance evidence-based solutions to prevent some of today’s biggest childhood health concerns (http://www.targetkids.ca). We have leveraged existing *TARGet Kids!* infrastructure, collaborations, research personnel and data management system to carry out this randomized controlled trial to determine whether wintertime high dose vitamin D supplementation of preschoolers reduces two common and costly child health outcomes, viral URTI and asthma exacerbations.

### Biochemistry and sources of vitamin D

Vitamin D is a fat soluble steroid with two clinically relevant metabolites: 25-hydroxyvitamin D and 1,25-dihydroxyvitamin D [9,10]. While 1,25-dihydroxyvitamin D is the active form, it is not reflective of vitamin D stores. Circulating levels of 25-hydroxyvitamin D are reflective of vitamin D stores and is commonly measured to determine vitamin D serum level [11].

Vitamin D can be synthesized in the skin by exposure to sunlight or can be ingested from dietary sources [9]. When exposed to solar ultraviolet B radiation the skin converts 7-dehydrocholesterol to vitamin D3 [12]. However, north of 42°N latitude, there is little production of vitamin D from the skin between November and March [13]. Therefore, in temperate climates where exposure to sunlight is limited for a significant part of the year, vitamin D levels fall dramatically during the winter and dietary sources of vitamin D become extremely important for avoiding deficiency [14]*.* Few foods aside from fatty fish, which are not regularly consumed by preschoolers, naturally contain vitamin D [1]. Data from the Canadian Community Health Survey and other sources suggest that the majority of vitamin D that preschoolers ingest is from vitamin D fortified cow’s milk which contains 100 IU of vitamin D per cup [15-17]. Unfortunately, the amount of cow’s milk that preschoolers typically drink is insufficient to receive enough vitamin D to avoid wintertime deficiency [3,17-20]*.* Further, efforts to increase cow’s milk consumption in this population may increase the prevalence of iron deficiency [21-25].

### Recommended vitamin D serum levels and supplementation for preschoolers

It is well established that 25-hydroxyvitamin D serum levels above 50 nmol/L in children are sufficient to prevent rickets [6]. Therefore, the American Academy of Pediatrics (AAP) suggests that 25-hydroxyvitamin D levels in children be above 50 nmol/L [4]. Data from adults suggest that serum levels above 75 nmol/L are required to minimize calcium resorption from bone and maximize intestinal calcium absorption [26]. Therefore, the Canadian Paediatric Society (CPS) suggests that optimal 25-hydroxyvitamin D level for children is above 75 nmol/L [5].

The recommended vitamin D dietary allowance (RDA) for children older than 1 year has been set at 600 IU/day by both Health Canada and the Institute of Medicine. Because children who consume less than 1000 ml of vitamin D fortified cow’s milk per day (which includes most preschoolers) [3,27] are unlikely to receive this amount of dietary vitamin D, the AAP recommends routine vitamin D supplementation of 400 IU/day for all children ingesting < 1000 ml/day of vitamin D-fortified formula or cow’s milk [4]. The CPS has no recommendation for routine vitamin D supplementation for Canadian children older than 1 year [5]. However, we have found that 56% of our population is receiving 400 IU/day of supplemental vitamin D [28,29]. To reflect the current AAP recommendation and common practice in our population, we chose 400 IU/day for the ‘standard dose’ arm.

### Low vitamin D levels in North American children

There is consistent evidence, from both single center studies [17,30,31] and national surveys [18,19,27], that North American children older than 1 year have vitamin D serum levels lower than AAP or CPS recommendations (see Additional file 1). Data from the 2001–2004 National Health and Nutrition Examination Survey (NHANES) indicated that 70% of children 1 to 11 years had vitamin D levels < 75 nmol/L [18,19]. Data from the 2007–2009 Canadian Health Measures Survey suggested that 51% of children age 6–11 years had vitamin D levels < 75 nmol/L. Studies of pre-school aged children from Boston (42°N), Toronto (43°N), St. John’s (47°N), Calgary (51°N), Edmonton (53°N) and Alaska (58-61°N) have all found significant rates of vitamin D deficiency in infants and toddlers using various definitions [15,17,30-32].

### Potential child health consequences of low vitamin D levels

Severe vitamin D deficiency (25-hydroxyvitamin D level < 25 nmol/L) results in rickets [1], an irreversible bowing of the long bones and deformity of the joints and teeth with well described long term implications for skeletal growth [11]. Important health policy recommendations including vitamin D fortification of cow’s milk and universal vitamin D supplementation of breast fed infants has dramatically reduced the prevalence of rickets in North America (estimated to be 2.9 cases per 100,000 children in Canada) [33]. The more recent observation that the vitamin D receptor (VDR) is expressed in many tissues in the body in addition to the skeletal and endocrine systems has suggested that vitamin D may be acting in other ways [9]. Population-based, retrospective cohort and case–control studies have suggested that less severe vitamin D deficiency (25–75 nmol/L) in children may be associated with several other adverse health outcomes [34-41].

### Viral upper respiratory tract infection

Viral upper respiratory tract infections (URTI) are the most common infectious disease in North America [42]. URTI is the most common reason for emergency department visits and unscheduled outpatient visits in Canada comprising 10% of emergency department visits for children under 10 years of age [2,43]. Preschoolers have the highest incidence of URTI of any age group, occurring 1–2 times per month per child during the winter and higher among children who attend daycare [42,44-49]. Influenza, RSV and adenovirus, which collectively comprise 25% of respiratory infections in children, are the most common viruses that lead to febrile illness, acute otitis media, outpatient visits and hospitalization [44,50]. Roughly 50% of preschoolers with URTI are brought to medical attention resulting in an additional outpatient physician visit every 2 to 3 months during the winter with 1% requiring hospitalization [44,51]. Several groups have estimated the direct and indirect costs of URTI in preschoolers to be between $261 and $276 per URTI with influenza being the most costly virus, contributing $809 per URTI [45,51]. The collective cost of URTI in children under 5 years of age has been estimated to be $1.8 billion annually in the US [52]. Evidence supporting a causal connection between low vitamin D serum levels and URTI comes from multiple sources.

#### Temporality

Both vitamin D levels and viral URTI show a remarkably similar seasonal oscillation. R. Edgar Hope-Simpson hypothesized that a “seasonal stimulus” must affect the pathogenesis of influenza and Cannel et al. hypothesized that this seasonal stimulus may be related to seasonal oscillation of vitamin D levels [53,54].

#### Biological plausibility

Basic science has uncovered the role of vitamin D on the innate immune system [55]. The primary site for human contact with respiratory viruses is the upper respiratory tract mucosa [56]. Vitamin D is constitutively converted to its active form 1,25-hydroxyvitamin D in respiratory epithelium [57]. The mucosa of the upper airway is protected from infection by a complex set of peptides which have direct antimicrobial properties and contribute to innate immunity [58]. These peptides include defensins and calethicidin which have direct antiviral properties [59]. Furthermore, respiratory tract macrophages are stimulated to produce these peptides *in vitro* by the presence of 25-hydroxyvitamin D and *in vivo* through vitamin D supplementation [60,61].

#### Epidemiologic association

Observational studies support an association between viral infections and vitamin D levels in both adults and children. A post hoc analysis of a 3-year RCT of vitamin D supplementation for bone loss in 208 post-menopausal African American women found that 26 patients in the placebo group vs. 8 in the intervention group reported having a URTI (P = 0.002) [62]. Based on data from NHANESIII, the odds of having a recent URTI in Americans 12 years of age or older was 25% higher for people with 25-hydroxyvitamin D < 75 nmol/L relative to those > 75 nmol/L [63]. Young male Finnish soldiers with 25-hydroxyvitamin D levels below 40 nmol/L had nearly double the number of absent days from duty due to respiratory infections than soldiers with levels above 40 nmol/L [64]. In a prospective cohort study of 198 adults in Connecticut U.S., 25-hydroxyvitamin D concentrations > 95 nmol/L were associated with a two-fold reduction in URTI over a single winter [65]. Based on data from a New Zealand birth cohort, infants with 25-hydroxyvitamin D levels in cord blood below 25 nmol/L were at 2-fold higher risk of viral respiratory tract infection at 3 months of age than infants with cord blood levels above 75 nmol/L [66].

#### Randomized controlled trial (RCT) evidence

To our knowledge, no RCT has examined the effect of vitamin D supplementation on health outcomes in preschoolers. Three RCTs have examined the effect of vitamin D supplementation on URTI, two in adults and one in older children. Li-Ng and colleagues randomized 162 adults to 2000 IU per day of vitamin D or placebo for 3 months during the winter in Long Island, NY U.S. and recorded the frequency of URTI symptoms (without laboratory viral confirmation) using a bi-weekly online questionnaire. They found no difference in the incidence of reported URTI between vitamin D and placebo groups (48 URTIs vs. 50 URTIs, p = 0.57) [67]. As the authors point out, the lack of effect may have resulted from a relatively small difference in follow-up vitamin D levels in the vitamin D (88 nmol/L) vs. placebo group (63 nmol/L). Recently, Laaski et al. randomized 164 male Finnish army recruits to 400 IU of vitamin D per day vs. placebo between October and March [68]. Their primary outcome, mean number of days absent from duty due to URTI in the vitamin D vs. placebo group, demonstrated a trend towards reduced absenteeism (2.2 days vs. 3.0 days, p = 0.096). However, the dose of vitamin D chosen (400 IU per day), may not have been sufficient to raise 25-hydroxyvitamin D levels enough to impact significantly on viral URTI (72 nmol/L in the intervention group vs. 51 nmol/L for placebo). In the third trial, Urashima and colleagues randomized 167 six to 15 year old schoolchildren to 1200 IU per day of vitamin D or placebo for four winter months in Tokyo, Japan [69]. Their primary outcome, laboratory confirmed Influenza A infection, showed a statistically significant reduction in the vitamin D group vs. control group (11% vs. 19%, p = 0.04). However, the authors did not measure baseline or follow-up vitamin D serum levels so it is unclear whether the positive effect was due to an increase in vitamin D levels. To maximize our likelihood of finding a treatment effect and keeping total vitamin D intake below the Tolerable Upper Intake Level as recommended by Health Canada (2500 IU/day for preschoolers) [70], we chose 2000 IU/day for the ‘high dose’ arm.

### Asthma

Asthma is the most common chronic illness of childhood. Preschoolers bear the highest burden of asthma which affects 13% of children under 5 years of age compared with 8% of children under 18 years in Ontario [71]. Asthma exacerbations are the most common non-surgical cause for hospitalization of children in Canada, costing the Canadian health care system over $300 million annually [71-74]. An association between vitamin D deficiency and asthma exacerbation was initially proposed to explain the observation that low vitamin D levels and asthma exacerbations are both more common in temperate climates and more common during the winter months [75,76]. Recent basic science and epidemiological research has supported a connection between vitamin D serum levels and asthma.

#### Biological plausibility

In vitro studies have demonstrated that the VDR is present in bronchial smooth muscle cells and that many asthma-associated genes are expressed following stimulation of lung tissue with vitamin D [77,78]. Case–control studies have found associations between asthmatic individuals and polymorphisms in the VDR and other vitamin D related genes [79-81].

#### Epidemiologic association

Cross-sectional data from NHANESIII found a strong correlation between airway resistance and vitamin D levels in adults [82]. An examination of incident vitamin D levels in a cohort of 1024 seven to ten year old American children with mild-to-moderate asthma identified that children with 25-hydroxyvitamin D levels below 75 nmol/L had increased odds of emergency department visits and hospitalizations relative to children with vitamin D levels above 75 nmol/L over a period of 4 years (OR 1.5, p = 0.01) [83]. Furthermore, two cross-sectional studies of American and Costa Rican children suggested that children with vitamin D levels below 75 nmol/L had increased airway resistance and increased use of inhaled and oral steroids relative to children with vitamin D levels above 75 nmol/L [84,85]. Whether these effects are due to vitamin D deficiency or are mediated through a vitamin D related reduction in viral URTIs is not clear.

#### Randomized controlled trial (RCT) evidence

To our knowledge, no RCT has examined the effect of vitamin D on asthma exacerbations in preschoolers. In the trial by Urashima and colleagues of vitamin D supplementation of Japanese schoolchildren, asthma exacerbations were measured as a secondary outcome [69]. They found a statistically significant decrease in asthma exacerbations in children receiving 1200 IU of vitamin D per day vs. placebo (2 vs. 12 of 167 children, RR 0.17, p = 0.006). Interestingly, the reduction in URTI was stronger among children with asthma than those without (RR 0.17, p = 0.006) suggesting that the reduction in asthma exacerbations may be mediated through reduced viral URTIs among vitamin D supplemented children.

The objective of this paper is to serve as the first step in knowledge dissemination of the DO IT Trial. The current paper outlines the study protocol, explains the rationale for the study design and selection of outcome measures and documents some of the methodological considerations.

### Study objectives and hypotheses

The *primary objective* is to compare the effect of high dose orally supplemented vitamin D (2000 IU/day) vs. standard dose vitamin D supplementation (400 IU/day) in achieving a reduction in laboratory confirmed viral URTI during the winter in healthy preschoolers 1 to 5 years of age. We hypothesize that preschoolers supplemented with 2000 IU/day will have a reduction in wintertime viral URTI.

*Secondary objectives* include comparing high dose (2000 IU/day) vs. standard dose (400 IU/day) vitamin D supplementation for the following secondary outcomes: a) specific viral infections including influenza, adenovirus and respiratory syncytial virus (RSV); b) asthma exacerbations in preschoolers with asthma or recurrent wheezing; c) direct and indirect economic costs associated with URTIs; d) vitamin D deficient serum levels as defined by the AAP and CPS. We hypothesize that the likelihood of these secondary outcomes will be lower in children supplemented with 2000 IU/day of vitamin D.

## Methods/design

### Participants

The parents of healthy children aged 1 to 5 years presenting to seven *TARGet Kids!* participating academic pediatric or family medicine group practices in Toronto, Canada for a scheduled well-child visit prior to viral season (September through November over four years) are being approached to participate. Practices have between 3 and 10 practicing physicians. We are excluding children with gestational age < 32 weeks as they are a high risk population for respiratory tract infection and asthma, children with chronic illness (except for asthma) on parental report which is known to interfere with vitamin D metabolism and increase the risk of respiratory infection, and those children with a sibling participating in the study to reduce clustering effects.

After parents of participating children provide informed consent, baseline characteristics are obtained using a parent-completed, standardized, data collection form based on questions used in the Canadian Community Health Survey (see Additional file 2: Baseline Data Collection Form) [86]. The following data is being collected: age, sex, birth weight, enrolment date, parents’ ethnicity, maternal age, education and health, duration of breastfeeding, bottle use, current and past vitamin D supplementation, dietary vitamin D intake using 3 day dietary recall, daily multivitamin use, influenza immunization status, screen viewing time, physical activity, outdoor time, and sun exposure. In addition, height, weight and skin pigmentation are being measured using standardized techniques. A venous blood sample is obtained to document baseline 25-hydroxyvitamin D levels.

### Study design

A multicentre pragmatic randomized controlled superiority trial is being conducted over four winters. As this trial's primary goal is to inform health policy and secondarily to contribute to an explanation of the causal relationship between vitamin D and child health outcomes, this trial has been designed along the pragmatic end of the pragmatic-explanatory continuum, as described by KT (co-author) and other leading trial methodologists [87]. Specifically, eligibility criteria, participant compliance, follow-up intensity, and primary analysis are following pragmatic approaches (“Does this intervention work under usual conditions?”); whereas, follow-up of outcomes are following approaches mid-way along the pragmatic-explanatory continuum (“Can this intervention work under ideal conditions?”). This protocol follows the 2013 SPIRIT guidelines (Defining Standard Protocol Items for Randomized Trials) [88,89]. Trial results will be reported according to the 2010 CONSORT guidelines for pragmatic trials [90].

### Intervention

**Figure 1:**
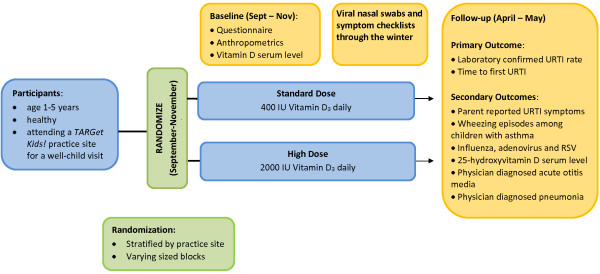
DO IT! Trial Schematic.

Children are randomly assigned to one of two groups: ‘standard dose’ of 400 IU/day vitamin D or ‘high dose’ of 2000 IU/day vitamin D (see Figure 1). A drop-based formulation (Kids Ddrops™ containing Vitamin D3) was chosen to facilitate ease of administration to young children. Parents of children in each group are instructed to administer 1 drop of the provided solution to their child by mouth once daily, at any time of day, each day from the time of enrolment (September-November) through the winter until follow-up (April-May) 4–8 months later. As 25-hydroxyvitamin D levels are reported to stabilize within 8 weeks [91,92], and respiratory viruses tend to circulate in Canada November through April [93], children are recruited September through November. The duration of the intervention (4–8 months) has been chosen to mimic the routine practice of vitamin D supplementation of preschoolers over the Canadian winter (see Table 1).

**Table 1:** Schedule of procedures, assessments, and interventions for the DO IT! Trial (based on the SPIRIT template)

	**Study period**				
	**Pre-randomization**	**Intervention**		**Post-intervention follow-up**	**Close-out**
**TIMEPOINT**	**Year 1**	**0**	**Year 1**	**Year 1**	
	***Sept – Nov***		***Sept – March***	***April – May***	
	**Year 2**		**Year 2**	**Year 2**	
	***Sept – Nov***		***Sept – March***	***April – May***	
	**Year 3 and 4**		**Year 3 and 4**	**Year 3 and 4**	**Year 4**
	***Sept – Nov***		***Sept – March***	***April – May***	***June – Aug***
**INTERVENTIONS:**					
**‘Standard dose’ 400 IU/day**		X	X		
**‘High dose’ 2000 IU/day**		X	X		
**PROCEDURES:**					
**Informed consent**	X				
**Eligibility screen**	X				
**Randomization**		X			
**ASSESSMENTS:**					
**Baseline survey data**	X	X			
**Anthropometrics**		X			
**25-hydroxyvitamin D serum level**			X	X	
**Laboratory-confirmed URTI**			X		
**Asthma exacerbations**			X		
**Follow-up survey data**				X	
**ANALYSIS**					X

Concomitant interventions prohibited include over-the-counter multivitamins which contain vitamin D, over-the-counter vitamin D preparations and prescription vitamin D. As this is a pragmatic trial, no specific strategies are introduced to improve adherence. To monitor adherence at the end of the trial, parents are asked to return bottles and the amount of vitamin D administered is calculated based on the volume of solution remaining [94]. No specific criteria are being used for discontinuation or modification of the interventions, as the doses of vitamin D are within the safe and recommended dosages for children [70].

Block randomization occurs by study site with blocks of varying sizes to ensure that group sizes are similar at the end of each block [88]. This is particularly important in a study of vitamin D and URTI, as date of enrolment (time) may be an important covariate. Sequence generation stratified by practice site was performed centrally at the Applied Health Research Centre (AHRC) at St. Michael’s Hospital using a computer random number generator; KT (biostatistician and co-author) generated the allocation sequence. Parents, attending physicians, laboratory personnel, and study personnel conducting the outcome assessments, data analysts and investigators are blind to the group allocation. Allocation concealment is achieved by having the Pharmacy Department prepare the vitamin D preparations in sealed, serially numbered bottles identical in appearance and weight, with the drops similar in consistency and taste. Group allocation will be concealed until the final data analysis is performed.

### Outcomes and measures

The *primary outcome* is the number of laboratory-confirmed viral URTIs per child over the winter months. Because of the wide variety of respiratory viruses and URTI illness presentations, signs and symptoms of viral URTI are non-specific and may not be reliably measured by parental report [95,96]. Therefore, in addition to parents of enrolled children completing a symptom checklist with each URTI (see Additional file 3: Symptom Checklist), parents are asked to obtain a nasal swab from their child. Nasal swabs done by parents have been shown to be as effective in detecting respiratory viruses as those obtained by health professionals but less invasive and better tolerated [97]. Parents are instructed to place the swab in provided viral transport media and refrigerate until couriered to the study laboratory (Mount Sinai Services) within 24 hours [44,98]. Respiratory viruses are identified using reverse transcriptase polymerase chain reaction (RT-PCR) technology. RT-PCR is performed on each sample which has a sensitivity of 95% and specificity of 100% for the identification of respiratory RNA viruses, which is more sensitive than viral culture and comparable to immunofluorescence [99]. Samples are tested for 18 common respiratory viruses including influenza A and B, adenoviruses, respiratory syncytial virus (RSV), picornaviruses (enteroviruses and rhinoviruses), coronovirus, metapneumovirus and parainfluenza virus using the ID-Tag™ RVP assay using the Luminex xMAP™ system (Cat# R019A0105, TM Bioscience Corp., Toronto, ON) [100]. This is the same assay and machine that is routinely used in the Ontario public health laboratories.

*Secondary outcomes* include parent reported URTI, specific viral agents that cause URTI (influenza, adenovirus and RSV), asthma exacerbations among children with asthma, physician-diagnosed otitis media and pneumonia, emergency department visits and hospitalizations, collected by monthly telephone call and confirmed by review of the child’s clinic medical record. Follow-up 25-hydroxyvitamin D serum level is measured to determine the vitamin D dose vs. serum response relationship. Finally, change in 25-hydroxyvitamin D serum level from baseline is determined to document that an improvement in health outcomes is mediated through an increase in 25-hydroxyvitamin D serum level.

Parent reported URTI symptoms are defined as two or more of fever (>38°C), cough, runny nose, sore throat, headache, vomiting, feels unwell, muscle aches, ear ache or infection, poor appetite, not sleeping well, cranky/fussy, low energy or crying more than usual from a validated parent completed symptom checklist (CARIFS) collected with the viral sample. CARIFS has been developed, validated and extensively used by our team and others [101,102]. A new URTI cannot commence until ≥ 3 symptom free days since the end of a previous URTI [44].

Asthma is defined as parental report of asthma plus confirmation from the child’s medical record of 2 or more episodes of wheeze requiring the prescription of inhaled asthma controlling medications [103]. An asthma exacerbation is defined as a wheezing episode in children with asthma as obtained from parent completed symptom checklist based on the International Study of Asthma and Allergies in Childhood (ISAAC) [103].

Blood is drawn by trained pediatric phlebotomists from the antecubital vein for determination of 25-hydroxyvitamin D serum levels. Specimens are sent to the Clinical Biochemistry Laboratory at the Mount Sinai Hospital with the study requisition, where they are processed according to standard procedures. Total 25-hydroxyvitamin D is measured from serum samples using a competitive two-step chemiluminescence assay which has been validated for measurement of 25-hydroxyvitamin D in children older than 1 year of age [104]. The specific instrument that is used to analyze all samples is a Diasorin LIAISON® 25-hydroxyvitamin D TOTAL [105]. This technique and instrument have been chosen to be consistent with national vitamin D surveys from both Canada and the United states [19,27]. Extensive testing and validation of this machine has been performed and has demonstrated an intraassay imprecision of 7.2% at a concentration of 213 nmol/L and an interassay imprecision of 4.9% at 32 nmol/L, 8.9% at 77 nmol/L and 17.4% at 213 nmol/L, values which are well within acceptable limits for biochemical measurements. During this study, the instrument is monitored using the UK DEQUAS external quality assessment scheme which is an internationally recognized vitamin D quality assessment protocol [106].

### Recruitment and retention

Since June 2008, over 4500 children age 1–5 years have been recruited through *TARGet Kids!* practices with collection of questionnaires (demographics, lifestyle factors) and anthropometric measures (height, weight and waist circumference). Since December 2008 venous blood samples have been collected from over 2500 children [107]. *TARGet Kids!* is now operating out of seven sites with over 100 children per month being recruited with phlebotomy. Therefore, recruitment of 750 children in September through November over 4 consecutive seasons for this study is feasible.

We expect that there will be a different spectrum of infections as respiratory virus incidence, distribution and severity tend to vary from year to year.

Strategies to achieve adequate recruitment include approaching eligible subjects during a well-child visit. Lists of children scheduled for a well-child visit are reviewed in advance. An information package is mailed to each family 2 to 4 weeks prior to the visit inviting them to participate. This allows parents time for consideration in advance and reduce coercion to participate. Parents are approached in person by the research assistant while registering for the clinic visit. At the initial visit, baseline survey data, anthropometric measures, and baseline vitamin D serum level are collected.

Strategies for retention include a monthly telephone call to encourage collection of nasal swabs and completion of symptom checklists. Every reasonable attempt is made to locate patients at follow-up. Topical anesthetic cream (EMLA or Ametop) is offered to minimize discomfort from venipuncture. Blood is drawn in the primary care physician’s office negating the need to attend a separate laboratory visit. Parents who have moved out of district are offered to visit The Hospital for Sick Children for repeat laboratory testing. Should these efforts fail to obtain a blood sample, a home visit for phlebotomy is offered. This is expected to occur in less than 10% of subjects.

### Follow-up data collection and data management

Parents of participating children receive a monthly telephone call by a research assistant reminding them to administer the vitamin D supplement daily and record the number of asthma exacerbations, emergency department visits, hospitalizations and missed work days. Parents are also be reminded to collect nasal swabs and complete symptom checklists. In addition, parents are asked to return to their child’s physician’s office in April or May to capture peak respiratory virus season. At the follow-up visit, blood is drawn for 25-hydroxyvitamin D level and a follow-up data collection form completed. The following potential co-interventions are also measured: influenza vaccination, dietary vitamin D intake using a 3 day dietary recall [86], over-the-counter vitamin and mineral supplementation (i.e., calcium containing multivitamins), herbal remedies (i.e., echinacea) and hours per week in daycare (see Additional file 4: Follow-up Data Collection Form). This methodology has been used successfully by our group and others [44,45,98].

To ensure high quality data collection, research assistants are trained in the accurate completion of questionnaires, anthropometric measurement and are registered pediatric phlebotomists as per standard *TARGet Kids!* operating protocol. Weight is measured using a precision digital scale (±0.025% SECA, Hamburg Germany), standing height is measured using a stadiometer (SECA, Hamburg Germany), and skin pigmentation is measured using a narrow-band reflectometer (Dermaspectrometer, Cortex Technology) [108-111]. Questionnaires and URTI symptom checklists have been pilot tested to ensure understandability and reduce incomplete responses [101]. Collected data is electronically entered on a daily basis by research assistants at each site into the study’s central database via a secure web-based data portal.

The Applied Health Research Centre (AHRC) of the Keenan Research Centre, Li Ka Shing Knowledge Institute of St. Michael’s Hospital houses this central database and is the data management centre for this study (under the direction of MM, co-author). AHRC employs state-of-the art web-based data management software: Medidata RAVE™ (5.6.3) by Medidata Solutions Inc. RAVE™ uses secure encrypted web-based data capture technology and is the data repository for data collected during this study. RAVE™ is an industry-leading electronic data capture and clinical data management system, with user configurable workflows, sophisticated case report form (CRF) design, complex edit checking, and customized security parameters. RAVE™ allows our research assistants to enter data remotely in real time to the central database from any of the practice sites. RAVE™ has extensive built in reporting capabilities, and data can be exported to standard formats for data analysis (e.g., SAS). Laboratory tests are directly uploaded to RAVE™ through a secure web portal. These features enable *TARGet Kids!* to be highly efficient and are vital to the success of this study.

### Sample size

The sample size was based on asymptotic methods for a likelihood ratio test assuming a Poisson distributed outcome and was confirmed by simulation studies. All sample size and power calculations assume a 5% Type I error probability (two-sided). If we assume an average of one URTI per month during a minimum of 4 winter months [42,44-49] among children receiving the standard dose of vitamin D, we would therefore expect an average of four URTI per child over the winter. A sample size of 300 per group would give 90% power to detect a reduction in the average number of URTI per winter of one URTI. We believe that, even one fewer URTI over the winter would be a clinically important outcome, especially to families with young children. Although the study is not powered to detect reductions of specific kinds of infections, a conservative estimate is that at least 25% of children will have one of RSV, adenovirus or influenza which collectively result in the greatest burden of illness in this population [44,50], this sample size gives 80% power to detect a 50% absolute reduction in this composite outcome. Preliminary *TARGet Kids!* data suggests an 80% retention rate [107,112]; therefore, to accommodate a 20% loss to follow-up, 375 children will be recruited to each group (750 total).

### Statistical analysis

Baseline characteristics will be summarized by appropriate descriptive statistics. Although randomization is expected to balance the covariates, variables that demonstrate, by chance, a potentially clinically meaningful imbalance, will be considered as possible adjusting covariates.

All outcomes will be analyzed following the *intention-to-treat* principle [113]. The primary analysis of the primary outcome will assess the effect of vitamin D supplementation on laboratory-confirmed URTI. Mean URTI rates (per child) will be computed for each group. A Poisson regression model will be used to make the statistical comparison between the groups. *Variable length of follow-up time* will be accounted for by using a suitable offset (logarithm of observation duration) in the Poisson model. If there is evidence of overdispersion, a negative-binomial model may be considered. The secondary analysis of the primary outcome will use survival methods to examine time to first URTI and the Andersen-Gill extension of the Cox model to analyze recurrent URTI events [114]. The rationale for conducting the trial over four seasons is to increase the chance of a sufficiently active viral season to assess a protective effect of the intervention. This will also allow us to examine the consistency of the effect over the four seasons as strains of viruses vary from year to year.

Analysis of secondary outcomes will use standard methods for continuous data (i.e., vitamin D level) using means, ANOVA and linear regression with and without adjustment for baseline group differences. The incidence of binomial secondary outcomes such as specific viral infections will be summarized descriptively. Since the incidence of some infections may be low, logistic regression analyses will only be performed to assess the treatment effect when there are sufficient events (over 30). Subgroup analyses will be conducted for children with asthma. Frequency of asthma exacerbations will be analyzed using the same methods as those used for the primary outcome.

### Economic analysis

A cost-effectiveness analyses will be conducted using data from this trial. A societal perspective will be employed to calculate both direct health service utilization costs as well as indirect costs to families of an URTI. The time horizon of the analysis will be limited to the follow-up of this trial in order to leverage direct data. Given the short time horizon, discounting of costs and outcomes will not be applied. This analysis will include the cost of ‘high dose’ vitamin D supplementation, physician visits, medications (antimicrobial and over-the-counter), hospital admissions, emergency department visits and laboratory testing abstracted from the child’s medical record and costs associated with lost income from parental work absenteeism and time out of daycare obtained by monthly telephone call using previously described techniques [45,74,115]. Standard, publicly available costing sources will be used to cost resource utilization parameters. Specifically, we will use cost sources such as the Ontario Health Insurance Programs (OHIP), Ontario Case Costing Initiative (OCCI) and standard Ministry of Health (MOH) reimbursements for diagnostic tests. Sex-weighted hourly wage rate will be derived from Statistics Canada Data. The cost-effectiveness analysis will estimate the cost per laboratory-confirmed URTI avoided. The net benefit regression approach will be used to determine each patient’s net benefit from treatment (NB_*i*_) based upon the data collected on resource use and lost parental productivity [116]. Graphs will be used to illustrate the incremental net benefit assuming varying willingness to pay. Each estimate of net benefit will be adjusted for potential confounders through regression. In its simplest form, net benefit regression involves fitting the following simple linear regression model: NB_*i*_ = β_0_ + β_TX_TX_*i*_ + ϵ_*i*_ where TX_*i*_ is the *i*^th^ person’s treatment indicator (TX_*i*_ = 1 for new treatment and 0 for usual care) and ϵ_*i*_ is a stochastic error term [117]. Parametric confidence intervals for incremental net benefit will be compared to results of non-parametric bootstrapping to characterize statistical uncertainty in the economic analysis.

### Ethical considerations

This study was granted ethics approval by The Hospital for Sick Children Research Ethics Board (REB File No.: 1000025147) on September 14, 2011 and was re-approved by the REB for one year ending in September 2015. This study has been registered as a clinical trial (http://www.clinicaltrials.gov, ID NCT01419262). Written informed consent will be obtained from parents of all child participants prior to any data collection (see Additional file 5: Consent Form). Parents will benefit by the provision of 4–8 months of vitamin D supplementation for their child free of charge. Children may directly benefit via the identification of viral etiology for URTI symptoms and through identification of vitamin D deficiency. The child’s pediatrician will receive viral test results as well as blood results and manage their patient according to national clinical guidelines [5]. While a placebo arm may be ethically justified given the lack of evidence supporting improved health outcomes with supplementation, it is unlikely to be feasible given that current AAP vitamin D guidelines recommend vitamin D supplementation for children older than 1 year and the majority of families in our population are following this recommendation [4,29].

A data safety and monitoring board (DSMB) was established and is composed of a pediatrician, an endocrinologist, an infectious disease expert and a biostatistician and is responsible for monitoring of adverse events. All adverse events will be graded for severity (mild to life threatening) and assessed for relationship to the study intervention. The DSMB (which will be blinded to group allocation) will review safety data between years 1 and 2 and will make decisions regarding unblinding of study groups and premature trial termination. As vitamin D dosages in this study are within the Tolerable Upper Intake Level as recommended by Health Canada for children older than 1 year of age (2500 IU per day for children 1–3 years and 3000 IU/day for children 4–8 years), risk of vitamin D excess is low [70]. In addition, other studies which have used vitamin D doses of up to 50,000 IU per week in children did not show evidence of vitamin D toxicity [118,119]. However, we will monitor for adverse events by ensuring that our participating physicians are aware of signs and symptoms of vitamin D toxicity including nephrolithiasis and hypercalcemia. Additionally, each participant will be assessed for vitamin D toxicity at follow-up through measurement of serum calcium, alkaline phosphatase and parathyroid hormone [6,120,121].

### Knowledge translation

Findings from this research will be disseminated directly to the physician participants and their patients. We will share our results at the annual meeting of all the *TARGet Kids!* practices (physicians, nurses, office staff), research team (investigators, research assistants, students), and policy leaders (representatives from Section of Community Paediatrics, Family and Community Practice, Ontario Medical Association, and parent representatives). Downstream dissemination to primary care physicians will occur through formal and informal venues at local levels, such as City Wide Paediatric Rounds, The Hospital for Sick Children Paediatric Update and those held by local physician groups. The results of this study will be shared with the academic community through peer-review publications and presentations at national and international conferences, locally through hospital rounds and presentations, and through our website http://www.targetkids.ca/. Messages will be relevant to professionals working in the fields of pediatrics, family medicine, endocrinology, infectious disease, nursing, dietetics, and public health. We will also share our findings with colleagues at the Ontario Agency for Health Protection and Promotion, the Centre for Effective Practice, the Maternal Infant Child and Youth Research Network (MICYRN), the Ontario Medical Association, the Canadian Paediatric Society and the American Academy of Pediatrics. Opportunities for media coverage will be sought using an experienced knowledge broker.

## Discussion

There is compelling evidence that young children in North America have vitamin D levels significantly lower than experts recommend. Basic science and epidemiological studies make the case that low wintertime vitamin D levels may be related to two common and costly child health outcomes: wintertime viral URTI and asthma exacerbations. The DO IT Trial is the first randomized controlled trial to investigate vitamin D related respiratory health outcomes in preschoolers. The results of this trial will make an immediate contribution by defining clinical practice standards for vitamin D supplementation for young children and provide an evidence base for national vitamin D guidelines.

The key strengths of this study protocol include a novel and important research question, the outcome of which could affect nearly every young child in Canada, rigorous methodology and an implementation strategy that leverages the efficiency of the only primary care research platform for young children in Canada: *TARGet Kids!*. The *TARGet Kids!* infrastructure and collaborations between child health researchers at the St. Michael’s Hospital, The Hospital for Sick Children and community health care providers make this team unique in Canada and position it ideally to carry out this trial on vitamin D supplementation of Canadian preschoolers and provides opportunity for truly integrated knowledge translation. Furthermore, a solid interdisciplinary team of primary care practitioners and highly qualified methodologists has been assembled with established expertise in preventive child health research, vitamin D deficiency, infectious diseases, laboratory medicine, health economics, clinical epidemiology and clinical trials methodology, analysis and data management to conduct this much-needed research.

Data from this RCT will provide essential evidence regarding wintertime vitamin D supplementation and important health outcomes in Canadian children. Potential downstream economic and health benefits of this trial to Canadian children, the health care system and society include reduced population wide morbidity and associated health care and societal costs. The current paper serves as an important step in the dissemination of the DO IT! Trial by outlining the study background, explaining some of the methodological considerations, and providing a detailed description of methods prior to reporting the results of the analyses.

## Competing interests

All authors declare that they have no competing interests.

## Authors’ contributions

All authors made substantive intellectual contributions to conception and design of this study and manuscript. JLM and CMB were the lead authors responsible for initial drafting of the manuscript. All others, especially PCP and CSB in the early revision stages, revised it critically for important intellectual content. KT ensured the accuracy of the statistical information. JSH ensured the accuracy of the economic information. All authors read and approved the final manuscript.

## Pre-publication history

The pre-publication history for this paper can be accessed here:

http://www.biomedcentral.com/1471-2431/14/37/prepub

## Supplementary Material

Additional file 1 **Appendix 1.** North American studies of low vitamin D including children > 1 year of age. Data demonstrating Vitamin D levels are lower than recommendations. Click here for file

Additional file 2 **Appendix 2.** Baseline Data Collection Form. This is the baseline data collection form being used in our study. Click here for file

Additional file 3 **Symptom Checklist.** This is the symptom checklist being used in our study. Click here for file

Additional file 4 **Follow-up Data Collection Form – Data linking sheet.** This is the follow-up data collection form being used in our study. Click here for file

Additional file 5 **PARENT/GUARDIAN RESEARCH CONSENT FORM (for participants 1–5 years).** Model consent form for our study. Click here for file

## *TARGet Kids!* Collaboration

Scientific Committee: Kawsari Abdullah, Laura Anderson, Catherine S. Birken, Cornelia M. Borkhoff , Sarah Carsley, Yang Chen, Matthew D’Ascanio, Mikael Katz-Lavigne, Kanthi Kavikondala, Grace Jieun Lee, Jonathon L. Maguire, Jessica Omand, Patricia C. Parkin, Navindra Persaud, Meta van den Heuvel, Weeda Zabih; Site Investigators: Jillian Baker, Tony Barozzino, Joey Bonifacio, Douglas Campbell, Sohail Cheema, Brian Chisamore, Karoon Danayan, Paul Das, Mary Beth Derocher, Anh Do, Michael Dorey, Sloane Freeman, Keewai Fung, Charlie Guiang, Curtis Handford, Hailey Hatch, Sheila Jacobson, Tara Kiran, Holly Knowles, Bruce Kwok, Sheila Lakhoo, Margarita Lam-Antoniades, Eddy Lau, Fok-Han Leung, Jennifer Loo, Sarah Mahmoud, Rosemary Moodie, Julia Morinis, Sharon Naymark, Patricia Neelands, James Owen, Michael Peer, Marty Perlmutar, Navindra Persaud, Andrew Pinto, Michelle Porepa, Nasreen Ramji, Noor Ramji, Alana Rosenthal, Janet Saunderson, Rahul Saxena, Michael Sgro, Susan Shepherd, Barbara Smiltnieks, Carolyn Taylor, Thea Weisdors, Sheila Wijayasinghe, Peter Wong, Ethel Ying, Elizabeth Young.
