# Supplementary material for: DO IT Trial: vitamin D Outcomes and Interventions in Toddlers – a TARGet Kids! randomized controlled trial
Source: BMC Pediatr. 2014 Feb 8;14:37. doi: 10.1186/1471-2431-14-37 (PMC3942179; doi:10.1186/1471-2431-14-37)
Supplement: Additional file 1 — Appendix 1. North American studies of low vitamin D including children > 1 year of age. Data demonstrating Vitamin D levels are lower than recommendations. [file 1471-2431-14-37-S1.pdf]

Appendix 1. North American studies of low vitamin D including children > 1 year of age

Author	Location	Year	Latitude	Age	Season	N	<50 nmol/L	<75 nmol/L
Kumar [16]	Nationally representative US sample (NHANES)	2001-2004	NA	1-21 years	NA	6275	9% *	70%
Mansbach [15]	Nationally representative US sample (NHANES)	2001-2006	NA	1-5 years	Winter for lower latitudes; summer for higher latitudes	1799	14%	63%
Weng[155]	Philadelphia, US	NA	40° N	6-21 years	All Seasons	382	25%	55%
Gordon [14]	Boston, US	2005-2007	42° N	8-24 months	All seasons	133	12%	40%
Gordon [29]	Boston, US	2001-2003	42° N	11-18 years	All seasons	307	42%	NA
Gessner [28]	Alaska, US	2001-2002	58-61° N	6-23 months	All seasons	133	11% *	31% †
Roth [12]	Edmonton, Canada	2003	52° N	2-8 years	Spring	35	17%*	N/A
Newhook [30]	St. John's, NL, Canada	2005-2006	47° N	0-14 years	Fall/Spring	48	35%	77%
Langlois [25]	Nationally representative Canadian (CHMS)	2007-2009	NA	6-11 years	All seasons	453	NA	51%
Maguire [2]	Toronto, Canada	2007-2008	43° N	24-30 months	Winter/spring	91	32%	82%
Stoian[17]	Calgary, Canada	2006	51° N	2-13 years	All seasons	1442	NA	39%

* < 37.5 nmol/L
† < 62.5 nmol/L
NA = not available
