# Supplementary material for: DO IT Trial: vitamin D Outcomes and Interventions in Toddlers – a TARGet Kids! randomized controlled trial
Source: BMC Pediatr. 2014 Feb 8;14:37. doi: 10.1186/1471-2431-14-37 (PMC3942179; doi:10.1186/1471-2431-14-37)
Supplement: Additional file 2 — Appendix 2. Baseline Data Collection Form. This is the baseline data collection form being used in our study. [file 1471-2431-14-37-S2.pdf]

Office use only
ID_______________
(write on page 2 now)
OHIP______________________

Appendix 2: Baseline Data Collection Form

Vitamin D Supplementation RCT

Participant Information

(This sheet to be stored separately from study data)

Date: _____________________2010
Month Day Year

1. Please provide contact information for you, your child, and your child's doctor. **You will only be contacted if your responses need to be clarified.**

   a) **Your name:** ________________________________ **Phone #:** _______ - _______ - __________
   (First) (Last)

   Your relationship to the child:
   - ☐ Biological mother
   - ☐ Biological father
   - ☐ Adoptive mother
   - ☐ Adoptive father
   - ☐ Other: _____________________

   b) **Your child's name:** ________________________________ **Phone #:** _______ - _______ - __________
   (First) (Last)

   ☐ **SAME phone number as above**

   c) **Your child's doctor's name:** Dr. _____________________________
   (Initial) (Last)

2. Your postal code: ___ ___ ___ ___ ___ ___

3. Your child's date of birth: _______________________________
   Month Day Year

4. Your child's gender:
   - ☐ Female
   - ☐ Male

ID

**Answer these questions for mother AND father** ➔	Mother	Father
5. What are the ages of the child's parents?	________years	________years
6. Are the child's parents currently employed?	☐ No<br>☐ Yes Is mother:<br>☐ Part time employed<br>☐ Full time employed<br>☐ On parental leave<br>☐ Other________________	☐ No<br>☐ Yes Is father:<br>☐ Part time employed<br>☐ Full time employed<br>☐ On parental leave<br>☐ Other_________________
7. What is the immigration status of the parents?	☐ Canadian Citizen<br>☐ Landed immigrant<br>☐ Refugee	☐ Canadian Citizen<br>☐ Landed immigrant<br>☐ Refugee

8. What is the immigration status of your child?
- ☐ Candian Citizen
- ☐ Landed Immigrant
- ☐ Refugee
- ☐ International adoptee

9. Are biological parents of the child:

**Biological Mother**	**Biological Father**
☐ White	☐ White
☐ Chinese	☐ Chinese
☐ South Asian (e.g. East Indian, Pakistani, Sri Lankan, etc.)	☐ South Asian (East Indian, Pakistani, Sri Lankan, etc.)
☐ Black	☐ Black
☐ Filipino	☐ Filipino
☐ Latin American	☐ Latin American
☐ Southeast Asian (e.g. Vietnamese, Cambodian, etc.)	☐ Southeast Asian (e.g. Vietnamese, Cambodian, etc.)
☐ Arab	☐ Arab
☐ West Asian (e.g. Iranian, Afghan, etc.)	☐ West Asian (e.g. Iranian, Afghan, etc.)
☐ Korean	☐ Korean
☐ Japanese	☐ Japanese
☐ Other (please specify)	☐ Other (please specify)
________________________________	________________________________

10. What is the highest level of education completed by mother? ☐ Public School ☐High school ☐ College/University

11. Which of the following best describes your child's living arrangements?
- ☐ Lives with 2 parents in the same household
- ☐ Lives with 1 parent only
- ☐ Lives alternating with 2 parents in different households
- ☐ Other— **Please explain** ____

12. Did your child's **biological mother** take any vitamins or supplements **during her pregnancy?**
- ☐ No
- ☐ Prenatal multi-vitamin _______ times per ____________ (day, week, month, year)
- ☐ Iron _______ times per ____________ (day, week, month, year)
- ☐ Vitamin D _______ times per ____________ (day, week, month, year)
- ☐ Other— **Please explain**____

13. Did your child's **biological mother** take any vitamins or supplements **while breastfeeding?**
- ☐ Mother did not breast feed
- ☐ No
- ☐ Prenatal multi-vitamin _______ times per ____________ (day, week, month, year)
- ☐ Iron _______ times per ____________ (day, week, month, year)

☐ Vitamin D _______ times per ____________ (day, week, month, year)
Other— **Please explain**____

Questions about your child's health

14. Where was your child born? ________________ ___________________
City Country

15. What was your child's birth weight? _______Pounds ________Ounces (OR _________Grams)

16. Children are considered to be full term if they are born at 37-40 weeks gestation. Was your child born full term?
☐ Yes
☐ No
If **NO**, what was your child's gestational age at birth
☐ 37-42 weeks gestation
☐ 32-36 weeks gestation
☐ <32 weeks gestation

17. Do you consider your child to be healthy?
☐ Yes
☐ No**— Please explain**____

18. Has **your child** been ill within the past month?
☐ Yes— **Please explain**____
☐ No

19. Has **your child** been diagnosed with any of the following conditions? Please check ALL that apply.
☐ Asthma
☐ Diabetes
☐ Fatty Liver
☐ Eczema or Atopic Dermatitis
☐ High Blood pressure
☐ High cholesterol
☐ Attention Deficit Hyperactivity Disorder
☐ Autism or autism spectrum disorder
☐ Learning problem
☐ Other-Please explain____

20. Does **your child** take any vitamins or supplements regularly?
☐ No
☐ Multivitamin _______ times per ____________ (day, week, month, year)
☐ Vitamin D _______ times per ____________ (day, week, month, year)
☐ Calcium _______ times per ____________ (day, week, month, year)
☐ Other—**Please explain**____

21. Does **your child** regularly take any prescribed medications?
☐ Yes **–** Which ones? ____
☐ No

22. Please check all non-prescribed medications or substances that your child has taken **in the past 2 weeks.**
☐ Cold/flu medication
☐ Other - Please explain____
☐ None
☐ Unsure

23 Did your child receive Vitamin D drops during their first year of life?
☐ Yes
☐ No **(SKIP TO QUESTION 43)**
☐ Unsure **(SKIP TO QUESTION 43)**

d) At **what age** did you **stop** giving the Vitamin D drops? _____________

e) How often did you give the Vitamin D drops? _______ times per ________ (day, week, month, year)

24. Is your child **currently** breastfeeding?

- ☐ Yes
- ☐ No— At what age did you stop breastfeeding? ________________

25. Which scenario **best describes** your child?
- ☐ My child received infant formula 80-100% of the time (was exclusively formula fed),
- ☐ My child received breast milk 80-100% of the time (was exclusively breastfed).
- ☐ My child received both breast milk and formula equally.

26. For how long has your child received infant formula? ________ months _________ weeks

27. Does your child have any food allergies, intolerances or food restrictions that have **been confirmed by your child's** doctor?
- ☐ Yes - **What are they?** ____
- ☐ No

28. Please specify **your child's diet for the past 3 days**. **Please check all that apply.**
- ☐ Breast milk
- ☐ Infant formula
- ☐ Red meat (beef, veal, pork, lamb, etc.)
- ☐ Poultry (chicken, turkey, duck, etc.)
- ☐ Fish (salmon, halibut, haddock, cod, tuna, etc.)
- ☐ Shellfish (lobster, crab, shrimp, etc.)
- ☐ Eggs
- ☐ Milk **☐ Skim ☐ 1% ☐ 2% ☐ Homo**
- ☐ Cheese
- ☐ Yogurt
- ☐ Margarine
- ☐ Honey
- ☐ Unsure
- ☐ Vegetarian: does not eat red meat, poultry, fish or shellfish
- ☐ Vegan: does not eat red meat, poultry, fish, shellfish, eggs, dairy or honey

29. **Circle how many cups of each drink your child has <u>currently in a typical day.</u> (1 cup=8 ounces=250 ml)**

Cow's milk	0	½	1	2	3	4	5+
Infant formula	0	½	1	2	3	4	5+
Other milk (soy, rice, goat etc)	0	½	1	2	3	4	5+

55. If your child drinks **"other milk"** (soy, rice, goat etc) what **kind is it?** ________________________________

Questions about <u>screen time</u> (time spent in a room with the TV, video/DVD on, or using a computer)

30. On the **LAST WEEKDAY** how many minutes did your child spend awake in a room with:
The television on: _________minutes
Videos or a DVD on: _________minutes
Playing the computer: _________minutes
Playing video games: _________minutes

31. On the **LAST WEEKDAY**, which meals did your child eat in a room with the television on:

Breakfast	☐Yes	☐No
Lunch	☐Yes	☐No
Dinner	☐Yes	☐No
A snack	☐Yes	☐No

32. On the **LAST WEEKEND DAY**, how many minutes did your child spend awake in a room with:
The television on: _________minutes
Videos or a DVD on: _________minutes
Playing the computer: _________minutes
Playing video games: _________minutes

33. On the **LAST WEEKEND DAY**, which meals did your child eat in a room with the television on:

Breakfast	☐Yes	☐No
Lunch	☐Yes	☐No

Dinner	☐Yes	☐No
A snack	☐Yes	☐No

Physical activity is any activity that increases your child's heart rate and makes your child get out of breath some of the time. It can be done in sports, school activities, playing with friends, or walking to school.

34. Over a typical/usual week, on how many days is your child physically active for a total of at least 60 minutes per day?
(Add up all the time your child spends in physical activity each day)
- ☐ None (zero days)
- ☐ 1 day
- ☐ 2 days
- ☐ 3 days
- ☐ 4 days
- ☐ 5 days
- ☐ 6 days
- ☐ 7 days

35. Is your child currently in a licensed daycare or preschool program?
- ☐ Yes – **Last week**, how many hours did your child attend daycare or preschool? ____________ hours
- ☐ No **If no go to question 76**

36. On a **TYPICAL WEEKDAY**, how much time does your child spend outside for 'recess' or 'unstructured free play **during daycare or preschool?** ______________ minutes

37. **Aside from time in daycare and preschool**, on a **TYPICAL WEEKDAY**, how much time does your child spend outside in 'unstructured free play'? ______________ minutes

Questions about sun exposure

38. How much time did your child spend outside last week?

<1h 1h 2h 3h 4h 5h 6h >7h

39. In the summer, how often does your child play outside for at least 15 min with minimal clothing (without a shirt on or wearing only bathing suit)?

Never 1d/week 2d/week 3d/week 4d/week 5d/week 6d/week Every day

40. When outside in the summer, how often does your child wear a hat?

Never 25% of the time 50% of the time 75% of the time Always

41. When your child is outside in the summer, how often do you apply sun block to your child's exposed skin?

Never 25% of the time 50% of the time 75% of the time Always

42. Vitamin deficiencies may arise as a result of lack of sunlight exposure. Does your child's biological mother usually wear a head covering?
- ☐ Yes
- ☐ No
