# Supplementary material for: DO IT Trial: vitamin D Outcomes and Interventions in Toddlers – a TARGet Kids! randomized controlled trial
Source: BMC Pediatr. 2014 Feb 8;14:37. doi: 10.1186/1471-2431-14-37 (PMC3942179; doi:10.1186/1471-2431-14-37)
Supplement: Additional file 3 — Symptom Checklist. This is the symptom checklist being used in our study. [file 1471-2431-14-37-S3.pdf]

Office use only
ID_____________

SYMPTOM CHECKLIST

			If Yes, please tell us	
**Symptom**	**Yes**	**No**	**Start Date** (YYYY/MM/DD)	**End Date** (YYYY/MM/DD)
Fever (>=38°C)	❑	❑	_ _ _ _/ _ _/ _ _	_ _ _ _/ _ _/ _ _
Coughing	❑	❑	_ _ _ _/ _ _/ _ _	_ _ _ _/ _ _/ _ _
Runny nose	❑	❑	_ _ _ _/ _ _/ _ _	_ _ _ _/ _ _/ _ _
Sore throat	❑	❑	_ _ _ _/ _ _/ _ _	_ _ _ _/ _ _/ _ _
Headache	❑	❑	_ _ _ _/ _ _/ _ _	_ _ _ _/ _ _/ _ _
Vomiting	❑	❑	_ _ _ _/ _ _/ _ _	_ _ _ _/ _ _/ _ _
Feels unwell	❑	❑	_ _ _ _/ _ _/ _ _	_ _ _ _/ _ _/ _ _
Muscle aches	❑	❑	_ _ _ _/ _ _/ _ _	_ _ _ _/ _ _/ _ _
Ear ache	❑	❑	_ _ _ _/ _ _/ _ _	_ _ _ _/ _ _/ _ _
Ear infection*	❑	❑	_ _ _ _/ _ _/ _ _	_ _ _ _/ _ _/ _ _
Poor appetite	❑	❑	_ _ _ _/ _ _/ _ _	_ _ _ _/ _ _/ _ _
Not sleeping well	❑	❑	_ _ _ _/ _ _/ _ _	_ _ _ _/ _ _/ _ _
Irritable, cranky, fussy	❑	❑	_ _ _ _/ _ _/ _ _	_ _ _ _/ _ _/ _ _
Low energy / tired	❑	❑	_ _ _ _/ _ _/ _ _	_ _ _ _/ _ _/ _ _
Crying more than usual	❑	❑	_ _ _ _/ _ _/ _ _	_ _ _ _/ _ _/ _ _

*Must be diagnosed by a physician
