# Supplementary material for: DO IT Trial: vitamin D Outcomes and Interventions in Toddlers – a TARGet Kids! randomized controlled trial
Source: BMC Pediatr. 2014 Feb 8;14:37. doi: 10.1186/1471-2431-14-37 (PMC3942179; doi:10.1186/1471-2431-14-37)
Supplement: Additional file 4 — Follow-up Data Collection Form – Data linking sheet. This is the follow-up data collection form being used in our study. [file 1471-2431-14-37-S4.pdf]

DO IT Trial: vitamin D Outcomes and Interventions in Toddlers
Follow-up Data Collection Form – Data linking sheet
(to be stored separately from study data)

ID String # ________________ Date ________________________

Child's date of birth ___________ Age _____(months) Child's Sex: Male [ ] Female [ ]

Home Telephone #___________________ Work/cell Tel # _____________________

Name caregiver interviewed ____

Relationship to child ____

ID String # ________________

1. How many days last week did your child receive the provided supplement (circle one)?

   0 1 2 3 4 5 6 7

2. Over the past 3 months has your child received any other supplement? Yes No

   2a. If yes, which one(s) ____

   2b. If yes, how many days per week did your child receive this supplement?

   1 2 3 4 5 6 7

3. How hard or easy has it been to give the provided supplement?

   Easy → Hard
   0 1 2 3

4. Did you child like taking the supplement? Yes No

5. If your child did not use the provided supplement each day: Why?

Takes too long	Yes	No
Too messy	Yes	No
My child didn't like it	Yes	No
Not convinced of the benefit of vitamin D supplementation	Yes	No
To hard to give it	Yes	No

6. Did your child experience any of the following while administering the supplement?

Coughing	Yes	No
Choking	Yes	No
Unhappy with the taste	Yes	No

For office use only

Height ____________ cm

Weight ____________ kg

BMI ____________ kg/m^2

Volume of solution remaining _____ml

Skin Type	Skin Color
I	White; very fair; red or blond hair; blue eyes; freckles
II	White; fair; red or blond hair; blue, hazel, or green eyes
III	Cream white; fair with any eye or hair color; very common
IV	Brown; typical Mediterranean skin
V	Dark Brown; mid-eastern skin types
VI	Black
