# Supplementary material for: DO IT Trial: vitamin D Outcomes and Interventions in Toddlers – a TARGet Kids! randomized controlled trial
Source: BMC Pediatr. 2014 Feb 8;14:37. doi: 10.1186/1471-2431-14-37 (PMC3942179; doi:10.1186/1471-2431-14-37)
Supplement: Additional file 5 — PARENT/GUARDIAN RESEARCH CONSENT FORM (for participants 1–5 years). Model consent form for our study. [file 1471-2431-14-37-S5.pdf]

PARENT/GUARDIAN RESEARCH CONSENT FORM (for participants 1-5 years)

DO IT Trial: vitamin D Outcomes and Interventions in Toddlers

Investigator(s):

Principle Investigator:

Dr Jonathon Maguire (416) 919-3462

Co-investigators:

Dr Catherine Birken	(416) 813-6933	Dr Mark Loeb	(905) 525-9140 x26671
Dr Patricia Parkin	(416) 813-6933	Dr Muhammad Mamdani	(416) 864-3037
Dr Tony Mazzuli	(416) 586-4695	Dr Jeffrey Hoch	(416) 864-6060 x2194
Kevin Thorpe	(416) 864-5776	Dr Reinhold Vieth	(416) 586-5920

Co-ordinator:

Marina Khovratovich (416) 813-2129

Purpose of the Research:

Your child's physician is a member of TARGet Kids! (Toronto Area Research Group) which is a network of child health researchers from SickKids and St. Michael's hospitals and community doctors dedicated to improving the health of young children.

With the aim of "health research for every child," this network will collect medical evidence on common health problems affecting urban Canadian children. We have a special focus on measuring the nutritional health of children 1 to 5 years of age. This is the first group in Canada to study children in community settings with a goal to promote wellness and prevent disease. We are inviting you and your child to participate in this exciting new TARGet Kids! study.

This study is examining the effect of vitamin D supplementation in toddlers. Low vitamin D during childhood is associated with several chronic medical conditions including low bone mass, type 1 diabetes, multiple sclerosis, asthma, and a number of cancers. Sixty to eighty percent of children in North America have insufficient vitamin D level. This study will determine if vitamin D supplementation reduces the number of colds and asthma attacks (if applicable) your child has during the winter season. We will also determine how much supplemental vitamin D is needed for young Canadian children to have optimal vitamin D levels.

Description of the Research:

If you agree to participate, when your child attends their next scheduled doctor's visit, we will ask you to complete a short questionnaire (approximately15 minutes total). We will record your child's height/ length and weight. You will be instructed to give your child the provided vitamin D supplement daily for 4 months. Vitamin D dosages in this study are safe and within the Tolerable Upper Intake Level as recommended by Health Canada for children. In April or May you will be asked to return to your doctor's office to find out your child's vitamin D level. A trained health professional experienced with pediatric blood collection will take a small blood sample from your child at that time. We may also collect additional health information on your child using an OHIP number.

Potential Harms, Discomforts or Inconvenience:

We will collect a small blood sample (3-5mL of blood) from your child's arm using a needle. There may be slight discomfort, bruising or redness that will usually disappear in a few days.

Blood collection is usually a quick process (about 5 minutes) and at other times it can require a little more time. While the amount of blood that will be collected is small, the impact that it will have in terms of helping sick children is immeasurable

Potential Benefits:
Your child may benefit from participating in this study by having his or her blood vitamin D level measured. Results of this test will be provided to your child's doctor with your permission. This could inform your doctor if your child might need an ongoing vitamin D supplement. In addition to knowing one has helped sick children across Canada and the world, participants themselves (and/or family member or friend) could potentially be a patient at SickKids or other paediatric health centre, and benefit directly from the results obtained from this study.

The major benefit of this study will be an accurate and reliable determination of how much supplemental vitamin D is needed to have vitamin D blood levels in the optimal range. This information would be very important to parents, children and health care providers across the country and internationally.

Confidentiality:
We will respect your privacy. No information about who your child is will be given to anyone or be published without your permission, unless required by law. For example, the law could make us give information about you if a child has been abused, if your child has an illness that could spread to others, if your child or someone else talks about harming themselves or others, or if the court orders us to give them the study papers.

SickKids Clinical Research Monitors, employees of the funders, or the regulator of the study may see your questionnaire responses or your child's blood test results to check on the study. By signing this consent form, you agree to let these people look at this information.

The data produced from this study will be stored in a secure, locked location. Only members of the research team (and maybe those described above) will have access to the data. This could include external research team members. Following completion of the research study, the data will be kept as long as required then destroyed as required by SickKids policy. Published study results will not reveal you or your child's identity.

We will give you a copy of this consent form.

Participation:
It is your choice to allow your child to take part in this study. You can stop at any time.

During this study we may create new tests, new medicines, or other things that may be worth some money. Although we may make money from these findings, we cannot give you or your child any of this money now or in the future because your child took part in this study.

Participation in research is voluntary. If you choose not to participate, you and your family will continue to have access to quality care at SickKids and at your child's doctor's office if needed. If you choose on behalf of your child to participate in this study you can take your child out of the study at any time. Again, you and your family will continue to have access to quality care at SickKids, St. Michael's Hospital and at your child's doctor's office if needed.

New information that we get while we are doing this study may affect your decision to take part in this study. If this happens, we will tell you about this new information. And we will ask you

again if you still want to be in the study. If your child becomes ill or is harmed because of study participation, we will treat your child for free.

Your signing this consent form does not interfere with your legal rights in any way. The staff of the studies, the hospital, and the doctor's office is still responsible, legally and professionally, for what they do.

Conflict of Interest
None of the people involved in this study have a conflict of interest. This means that they will not benefit personally or financially from this study.

Sponsorship:
Funding for this TARGet Kids study is provided by the Canadian Institutes of Health Research (CIHR)

Future Research:
In the future, our research team may approach you to participate in other studies with the aim of improving children's health. The research will be explained to you and your consent will be asked for at that time.

Consent :
By signing this form, I agree that:
1) You have explained these studies to me. You have answered all my questions.
2) You have explained the possible harms and benefits (if any) of these studies.
3) I understand that I have the right to refuse to let my child take part in the study. I also have the right to take my child out of the study at any time. My decision about my child taking part in the study will not affect my child's health care.
4) I am free now, and in the future, to ask questions about the study.
5) I have been told that my child's questionnaires, blood test results and medical records will be kept private except as described to me.
6) I understand that no information about my child will be given to anyone unless required by law.
7) I understand the publication of the results from this study will not identify me or my child in any way.

I agree, or consent, that my child___________________ may take part in this study.

______________________________ Printed Name of Parent/Legal Guardian	________________________ Parent/Legal Guardian's signature	______ date
______________________________ Printed Name of person who explained consent	________________________ Signature of Person who explained consent	______ date
______________________________ Printed Witness' name (if the parent/legal guardian does not read English)	________________________ Witness' signature	______ date

If you have any questions about the TARGet Kids study, please call Dr Jonathon Maguire at (416) 919-3462. If you have questions about your child's rights as a subject in a study or injuries during a study, please call the Research Ethics Manager at 416-813-5718.
